# All-Printed Flexible Memristor with Metal–Non-Metal-Doped TiO_2_ Nanoparticle Thin Films

**DOI:** 10.3390/nano12132289

**Published:** 2022-07-03

**Authors:** Maryam Khan, Hafiz Mohammad Mutee Ur Rehman, Rida Tehreem, Muhammad Saqib, Muhammad Muqeet Rehman, Woo-Young Kim

**Affiliations:** 1 Department of Electronic Engineering, Jeju National University, Jeju 63243, Korea; maryamkhan93@stu.jejunu.ac.kr (M.K.); mutee1990@jejunu.ac.kr (H.M.M.U.R.); saqibmuhammad@jejunu.ac.kr (M.S.); 2 H.E.J. Research Institute of Chemistry, International Center for Chemical and Biological Sciences, University of Karachi, Karachi 75270, Pakistan; ridatehreem595@gmail.com

**Keywords:** Cr-N-doped TiO_2_ nanoparticles, memristor, all-printed, flexible, forming-free

## Abstract

A memristor is a fundamental electronic device that operates like a biological synapse and is considered as the solution of classical von Neumann computers. Here, a fully printed and flexible memristor is fabricated by depositing a thin film of metal–non-metal (chromium-nitrogen)-doped titanium dioxide (TiO_2_). The resulting device exhibited enhanced performance with self-rectifying and forming free bipolar switching behavior. Doping was performed to bring stability in the performance of the memristor by controlling the defects and impurity levels. The forming free memristor exhibited characteristic behavior of bipolar resistive switching with a high on/off ratio (2.5 × 10^3^), high endurance (500 cycles), long retention time (5 × 10^3^ s) and low operating voltage (±1 V). Doping the thin film of TiO_2_ with metal–non-metal had a significant effect on the switching properties and conduction mechanism as it directly affected the energy bandgap by lowering it from 3.2 eV to 2.76 eV. Doping enhanced the mobility of charge carriers and eased the process of filament formation by suppressing its randomness between electrodes under the applied electric field. Furthermore, metal–non-metal-doped TiO_2_ thin film exhibited less switching current and improved non-linearity by controlling the surface defects.

## 1. Introduction

Data storage with high scalability has long been in demand and advancements in this area seem to be a never-ending research field. NAND flash memory devices have reached the limit of scalability due to which an alternative should be found [1]. A scientist at HP labs proposed an alternative to existing memory devices by introducing a new memory element known as a “memristor” in their laboratory [2,3,4,5,6,7,8,9,10]. A memristor is a memory device with two resistance states, high resistive state (HRS) and low resistive state (LRS), that can be switched interchangeably (SET/RESET) depending on the polarity and magnitude of applied voltage [11,12,13]. Memristors have drawn massive attention since their advent in 2008 owing to their potential applications as neuromorphic devices and unique characteristics such as low power losses, simple structure, small size, high data density, flexibility, etc. [14,15,16,17,18]. Memristors can be fabricated by using state-of-the-art and advanced technologies and its device size can vary from the nanometer (nm) to micrometer (µm) scale. Among these fabrication technologies, memristors fabricated from printing techniques have a larger size than those fabricated through solid state techniques [19]. However, printing technology holds various other advantages including low material wastage, fast prototyping, and high controllability. Furthermore, printing technology is also compatible with stretchable, flexible and transparent substrates [20,21,22]. Prototyping of a memristive device is easy because it has a simple structure with only three layers, including a tope electrode, functional layer and bottom electrode. Various metals and even other highly conductive materials such as graphene can be used as the electrodes; however, the sandwiched layer of a memristor has been a diverse topic of research for several years now to decide which active material can provide the best set of electrical, mechanical, chemical and physical properties. 

Materials ranging from transition metal oxides (TMOs), two-dimensional (2D) materials, polymers, inorganic semiconductors, natural materials and their composites have been explored to find memristive properties in them [13,23,24,25]. It is difficult to find all desired characteristics of a memristor in a single material, such as high switching ratio, flexibility, high electrical/mechanical endurance, low power consumption, fast switching speed, small device size, and high retention time. Therefore, it is necessary to engineer the defects of those functional materials that have shown promising characteristics of a memristor along with finding new materials. Doping a functional material is a useful tool to control its characteristics and therefore recently, Kim et al. [26] increased the non-linearity and reduced the switching current of silicon nitride (SiN) with high doping of Si substrate. Wu et al. [17] reduced the forming voltage and initial resistance of a HfO_2_-based memristor by doping the functional layer with aluminum (Al) atoms. These desirable results were achieved due to the formation of relatively weaker bonds between HfO_2_ and Al (Hf-Al-O) that are easy to break as compared to strong oxygen vacancies. Doping of HfO_2_ with Al also helped in the stable transition between the two resistive states as compared to its non-doped counterpart i.e., HRS-LRS and LRS-HRS. Sokolov et al. [27] enhanced the functional groups on the surface of graphene quantum dots (GQDs) by doping them with N. Their results verified that charge distribution was less in their memristive device before doping, i.e., lower electronegativity (zeta potential) as compared to doped GQDs. They also showed that transition switching between resistive states of the undoped device was less stable than the doped device. Zhu et al. [28] engineered the functional layer of HfO_2_ by doping it with Al to achieve robust and uniform memristive behavior. The obtained results clearly exhibited a more stable cycle endurance owing to improved surface roughness and control over the formation/rupture of filaments. They also showed that varying doping interfaces affects the performance of the device in a different way. Liu et al. [29] improved the robustness and power losses of a tin oxide (SnO_2_) memristor by doping it with bismuth (Bi). The Bi-doped device had a remarkably smaller transition voltage, compliance current (CC) and operating current. Doping the active layer with 4.8% Bi resulted in a massive reduction in operating power (16 µW) by two orders of magnitude as compared to a non-doped device. The volume of conductive filament (CF) in the doped memristor was much smaller than the undoped device; hence, the voltage required to form and rupture CF was also small. 

From the above discussion, it is verified that engineering the functional material has far-reaching good effects on the performance of a memristor. Hence, now we will discuss the potential effect of doping titanium dioxide (TiO_2_) with a variety of materials because TiO_2_ is the pioneer and leading functional material as far as the performance of a memristor is concerned. Yan et al. [30] used Ag-cluster-doped titanium dioxide (TiO_2_) and achieved enhanced performance of a TiO_2_-based memristor. The transition from short-term memory to long-term memory was successfully controlled with the help of doping self-assembled Ag clusters for reliable analog switching. Yuan et al. [31] studied the effect of doping a TiO_2_-based nanoscale memristor with copper (Cu) and also presented its mathematical model to form a chaotic circuit. Doping a TiO_2_ memristor with Cu resulted in achieving highly stable and symmetrical I-V characteristic curves with better rectification behavior. Kim et al. [32] doped titania with sodium (Na) by using the highly dependable technique of atomic layer deposition (ALD) and successfully reduced the major problem of cross talk between adjacent memory cells faced by all memristive devices. The resulting device exhibited an electroforming free bipolar resistive switching behavior with negligible sneak current of less than 100 pA. Crossbar arrays of Na-doped TiO_2_ memristors also achieved an accuracy of >99.1% for image recognition. Yu et al. [33] doped the nanorods of TiO_2_ with N to achieve a tunable nonvolatile memory for neuromorphic computing with the ability of mimicking the human brain in future. It was shown that N doping resulted in improved resistive switching characteristics such as endurance of over 8000 electric cycles due to the creation of additional anion vacancies in the functional layer of TiO_2_. Doping with N also helped in diversifying the balance between the creation and diffusion of these vacancies. Furthermore, the N-doped device exhibited the ability to be modulated with a pulse of short duration, hence resulting in a fast response time for mimicking a biological synapse.

In this study, we have doped the nanoparticles (NPs) of TiO_2_ with chromium (Cr) and nitrogen (N) by using the solution–gel method. The memristive behavior of doped TiO_2_ NPs was determined by sandwiching them between two Ag electrodes in the configuration of Ag/Cr-N-doped TiO_2_/Ag, which has not been reported on before. The resulting device exhibited enhanced performance in its switching ratio, endurance cycles, operating voltage, mechanical flexibility, and retention time. Both doping elements provided additional trap states for charge carriers to retain stored data for a longer time and lowered the energy barrier for the charge carriers to migrate from anode to cathode with a smaller magnitude of voltage supply. This all-printed and flexible doped TiO_2_ memristor is proposed to be the next-generation memory device owing to its enhanced performance and potential to be used for wearable electronics.

## 2. Experimental Section

### 2.1. Materials 

All the chemical and reagents used in this study were purchased from Sigma-Aldrich (Inc. St. Louis, MO, USA) and used without further purification. The chemicals and reagents used in this study were titanium isopropoxide, TTIP (Ti{OCH(CH_3_)_2_}4) 97%, acetic acid (CH_3_COOH) 99.8–100.5%, ethylene glycol (C_2_H_6_O_2_) ≥ 99%, urea (CO{NH_2_}) 99–100.5%, propanol HPLC grade (C_3_H_8_O) ≥ 99.5%, nitric acid (HNO_3_) 65%, chromium nitrate nonahydrate (Cr(NO_3_)_3_·9H_2_O) 98%, hydrochloric acid (HCl) ≥ 37%.

### 2.2. Synthesis of Cr-N Doped TiO_2_ NPs

An amount of 50 mL propanol was taken in a beaker and 6.65 mL TTIP was added to it with stirring. After 10 min of constant and vigorous stirring, 2 mL acetic acid was added to the solution, followed by temperature elevation to 70 °C. Then, 2 mL Ethylene glycol was added to lower the temperature, followed by 15 min stirring. This solution was termed solution A. The solution for nitrogen doping was then prepared by dissolving 0.1285 g urea in 10 mL distilled water. Next, 0.5 M HNO_3_ was added dropwise to set the solution pH at 2. This was completed to avoid precipitation of hydroxide ions. To prepare the solution for chromium doping, 10 mL distilled water was added in a beaker followed by addition of chromium nitrate and 2 mL 0.12 N hydrochloric acid. To this solution, 10 mL propanol was added with vigorous stirring. Both the dopants’ solutions were mixed, and this was termed as solution B. Solution B and solution A were mixed. This new solution was stirred for another 15 min at 500 rpm. The resulting mixture was dried in an oven at 100 °C for eight hours. The obtained dried particles were crushed and washed with 2 solvents (firstly with ethanol and then with distilled water) three times each. Washed particles were again dried to remove solvents and were then calcined at 450 °C for 2 h and 45 min.

### 2.3. Memristor Fabrication

**Figure 1:**
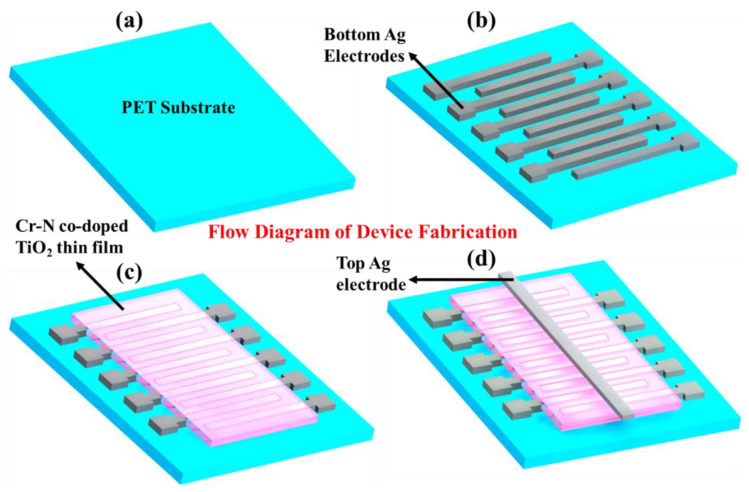
Flow diagram of Cr-N doped TiO_2_ NPs memristor. (**a**) Flexible and transparent bare PET substrate. (**b**) Bottom Ag electrodes patterned through advanced printing technology of reverse offset. (**c**) Functional thin film of Cr-N-doped TiO_2_ deposited by EHD atomization. (**d**) Final schematic diagram of doped TiO_2_ memristor after patterning top Ag electrode through EHD patterning.

An array of ten bottom Ag electrodes was patterned on the flexible Polyethylene Terephthalate (PET) substrate by using the advanced fabrication technology of reverse offset. Details of this printing technique can be found in our previous research work, but a brief description is provided here too [34]. Reverse offset is a direct contact method for patterning electrodes onto the desired substrate with the help of a roller and cliché. A cylindrical roller containing Ag ink on its surface was rolled over the cliché with the embedded patterns of desired electrodes. These patterns were then transferred onto the desired flexible PET substrate followed by sintering at 110 °C for 30 min. This method was preferred over other printing technologies owing to its unique features such as high resolution (<1 um), low resistivity and high accuracy. A thin film of Cr-N-doped TiO_2_ was deposited on the patterned Ag electrodes with the help of electrohydrodynamic atomization (EHDA) printing technology owing to its high controllability and ability to produce highly uniform thin films. Details about this printing technique can also be found in our previous report [35]. The fabrication of the doped TiO_2_ memristor was completed by depositing a top Ag electrode by using electrohydrodynamic (EHD) patterning. A complete flow diagram of the device fabrication process is shown in Figure 1. 

### 2.4. Characterization

The electrical characterizations were performed in an ambient environment at room temperature by using the semiconductor device analyzer (Agilent B1500A, KEYSIGHT, Santa Rosa, CA, USA). The morphological and structural studies of Cr-N-doped TiO_2_ NPs were conducted by field emission scanning electron microscopy (FE-SEM) (TESCAN, MIRA3, Oxford, Czech Republic), while elemental analysis to confirm the doping of chromium and nitrogen into the crystal structure of TiO_2_ was performed through EDX spectroscopy. Dynamic light scattering (DLS) using a Zetasizer Nano series (Malvern Panalytical, Malvern, UK) instrument was used to determine the surface charge. A Thermo Scientific Evolution 300 UV-VIS Spectrophotometer (Thermo Fisher Scientific, Waltham, MA, USA) was used for optical analysis. FTIR analysis was carried out by forming a KBr pallet and analyzing this through Broker Vector 22 (Ettlingen, Germany). XRD patterns were obtained on by Benchtop Powder X-ray Diffraction Instrument (Rigaku, Tokyo, Japan). InVia Raman Microscopy by (Renishaw; Wotton-under-Edge, UK) was utilized for obtaining RAMAN scattering with excitation laser at 514 nm. Mechanical bending was performed by using a custom-made testing machine. 

## 3. Results and Discussion

### 3.1. Characterization of Cr-N Doped TiO_2_ NPs

#### 3.1.1. Raman Analysis

**Figure 2:**
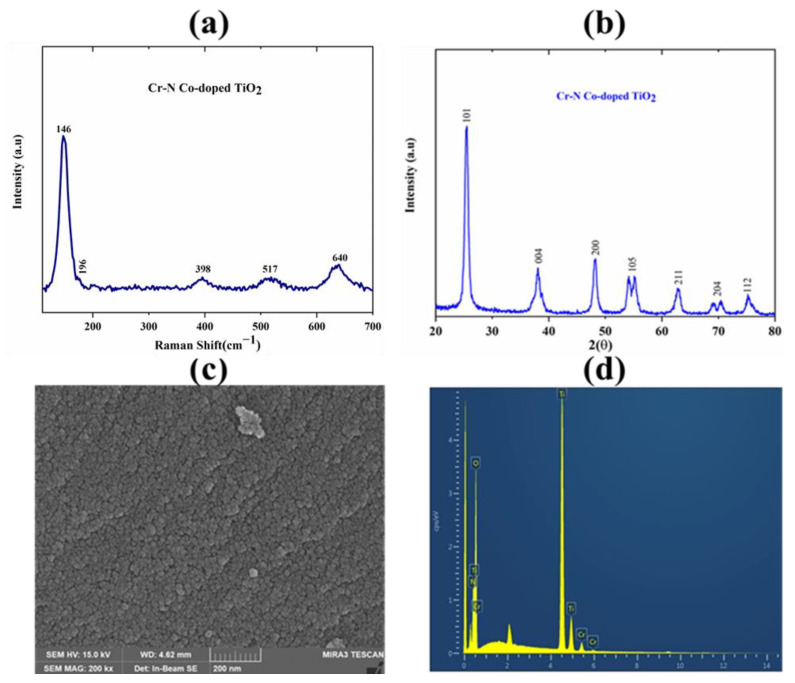
Material characterizations: (**a**) Raman scattering showing active optical phonon modes relevant to anatase structure of Cr-N-doped TiO_2_ NPs. (**b**) X-ray diffraction patterns indicating the single-phase anatase crystalline structure. (**c**) FESEM image of Cr-N doped TiO_2_ NPs showing homogeneous crystalline shape and size. (**d**) EDS spectra displaying the presence of chromium and nitrogen in crystalline matrix of TiO_2_.

There are two more common crystalline structures of TiO_2_: rutile and anatase. To study the nature of the crystalline structure of Cr-N-doped TiO_2_ NPs, Raman analysis was carried out. Raman analysis showed that Cr-N-doped TiO_2_ NPs synthesized in this study possess anatase structure. There are six Raman active optical phonon modes observed as shown in Figure 2a, denoting E_g_ (146 cm^−1^), E_g_ (196 cm^−1^), B_1g_ (398 cm^−1^), A_1g_ + B_1g_ (517 cm^−1^), and E_g_ (640 cm^−1^). These Raman active optical phonon modes confirm the formation of the anatase structure of as-synthesized Cr-N doped TiO_2_ NPs while the absence of noticeable phonon modes at 238 cm^−1^ and 445 cm^−1^ also confirm that it is not the rutile structure [36].

#### 3.1.2. XRD Analysis

X-ray diffraction analysis was carried out to confirm the crystalline nature and crystalline phase of the as-synthesized Cr-N-doped TiO_2_ NPs. Figure 2b shows the obtained XRD patterns with their corresponding atomic planes in accordance with JCPDS card No. 00-021-1272. The characteristic XRD peaks are displayed at 2θ = 25.54°, 38.08°, 48.28°, 54.16°, 62.76°, 70.44°, and 75.2°, indicating their corresponding planes (101), (004), (200), (105), (211), (204), and (112), respectively. The obtained XRD result confirmed the single-phase anatase structure of TiO_2_ and ruled out the mixture of rutile and anatase.

#### 3.1.3. FESEM Analysis

The morphology and homogeneity in size of as-synthesized Cr-N-doped TiO_2_ NPs was analyzed by FESEM. Figure 2c shows an almost spherical shape and homogeneous size as it is evident that there is no notable difference observed. Figure 2d displays the EDS spectra of Cr-N-doped TiO_2_ NPs that confirms the incorporation of chromium and nitrogen into the crystalline matrix of TiO_2_.

#### 3.1.4. FTIR Analysis

**Figure 3:**
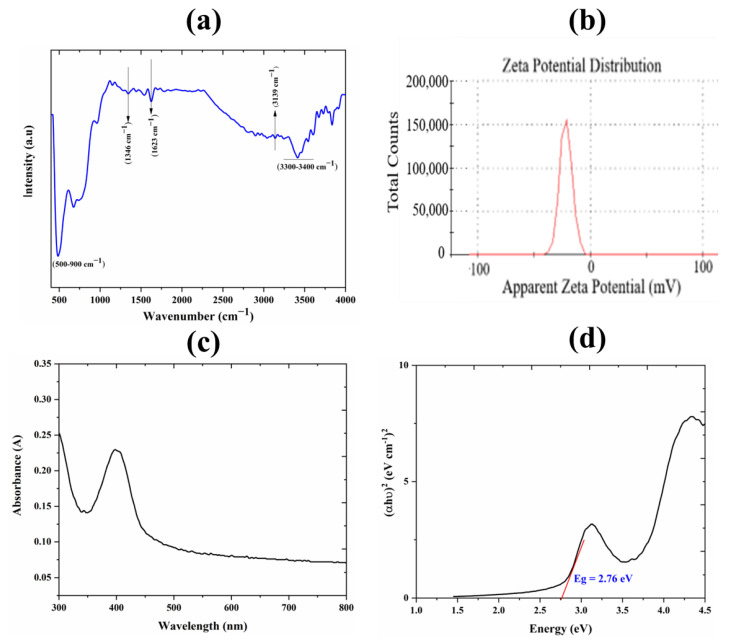
(**a**) FTIR spectra of Cr-N-doped TiO_2_ NPs corresponding the characteristic bonds. (**b**) Surface charge determination by zeta potential analysis showing anionic charge (−21.7) on surface of NPs. (**c**) UV-Vis absorbance spectra of Cr-N doped TiO_2_ NPs showing maximum absorbance in visible range at 400 nm. (**d**) The band gap energies calculated via Tauc Plot showing Eg = 2.76 eV.

FTIR analysis as shown in Figure 3a further investigates the chemical analysis of Cr-N-co-doped TiO_2_. The absorption peaks from (500–900 cm^−1^) exhibited the Ti-O-Ti bonds and Cr-O stretching bond vibrations. The absorption band at 1346 cm^−1^ shows the Ti-O-N-Ti bond, and the absorption peak at 1623 cm^−1^ indicates the stretching vibration of O-H and H_2_O. The absorption band at 3139 cm^−1^ is attributed to the N-H stretching vibrations, while a relatively broader peak from (3300–3400 cm^−1^) corresponds to the O-H stretching vibrations. These characteristic absorption peaks are in good agreement with the previous reported results of doped TiO_2_ [37,38].

#### 3.1.5. Zeta Potential Analysis

Zeta potential analysis has been carried out to determine the surface charge onto the surface of Cr-N-doped TiO_2_ nanoparticles, which may be very helpful to understand the mechanism of the resistive switching device. Figure 3b shows that Cr-N-doped TiO_2_ NPs possess anionic charge (−21.7) on their surface and demonstrated stable characteristics by retaining disaggregation for a longer period.

#### 3.1.6. UV-Vis Analysis

The UV-Vis spectroscopy technique was used to obtain the absorbance spectra and to determine the band gap of Cr-N-doped TiO_2_ NPs, as shown in Figure 3c. It has been observed through the obtained absorbance spectra that maximum absorbance was in a longer wavelength or visible region, i.e., almost at 400 nm. This is mainly due to the doping of Cr and N into the crystalline matrix of TiO_2_; otherwise, undoped TiO_2_ does not show absorbance at a longer wavelength [39,40]. Furthermore, the bandgap of Cr-N-doped TiO_2_ was determined through a Tauc plot of *(αhυ)^2^* vs. *hυ* as shown in Figure 3d. The determined bandgap was 2.76 eV, whereas the undoped TiO_2_ shows 3.2 eV, which has confirmed that doping of Cr and N lowered the bandgap of TiO_2_.

### 3.2. Electrical Characterization

**Figure 4:**
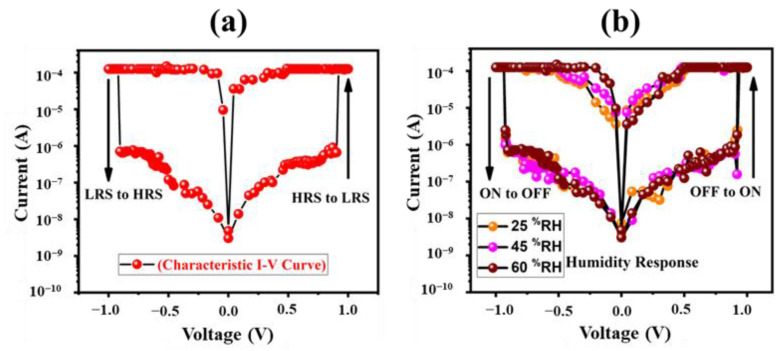
Electrical characteristics of metal–non-metal-doped TiO_2_ based memristor. (**a**) Signature graph of memristor showing its bipolar resistive switching behavior in the operating voltage range of ± 1 V. (**b**) Effect on electrical characteristics of flexible memristor under the varying relative humidity levels of 25%, 45% and 60%, respectively.

The fabricated device showed the signature I–V characteristic graph of a bipolar memristor with a pinched hysteresis loop around the origin, as illustrated in Figure 4a. Positive voltage was applied to the anode while the cathode was grounded. This memory device was initially in its high resistance state (HRS) before the application of an external voltage supply; however, it switched to a low resistance state with a voltage sweep of ±1 V across the anode and cathode. The obtained I–V curve was symmetric around the zero axis because of the use of the same material (Ag) as the top and bottom electrode. Current compliance (CC) of 10 mA was set to protect the device from sudden breakdown due to an excessive heating effect. The characteristic I–V curve of our device showed that current increased non-linearly with increasing voltage while retaining its HRS until the applied voltage reached its threshold (Vth) value of Vth ~0.9 V. The HRS (Off state) abruptly changed to LRS (On state) after reaching Vth. Similar behavior was observed in the region of the negative axis where the device switched back to LRS from HRS after reaching Vth ~−0.9 V. It can be depicted from this information that digital data can be stored/written in the form of binary digits at Vth ~0.9 V and stored data can be removed by applying a reverse voltage of ~−0.9 V. After successfully verifying the bi-polar memristive behavior in a Cr-N -doped TiO2 memory device, we evaluated the ef-fect of humidity on its performance, and the obtained results are shown in Figure 4b. Humidity had little or no effect on the nonvolatile memristor in the range of 25–60%RH, which shows the ability of our device to be used in highly humid conditions.

**Figure 5:**
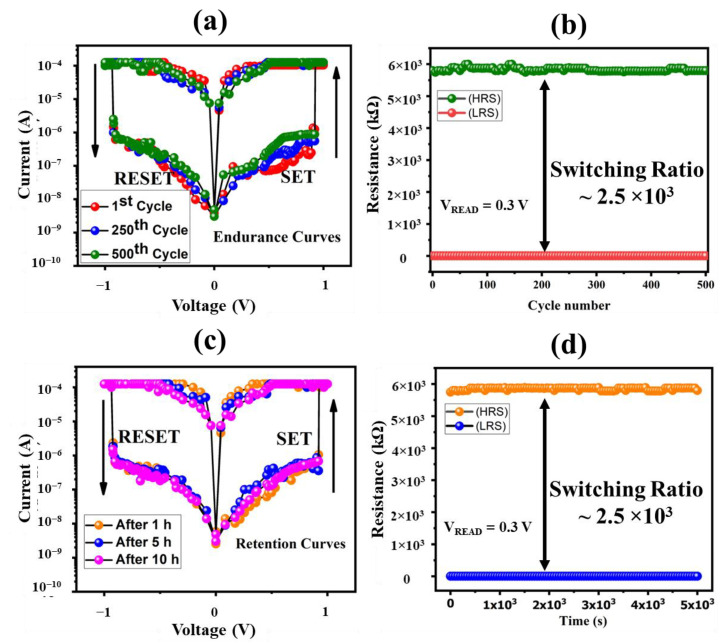
Electrical characteristics of metal–non-metal-doped TiO_2_ based memristor. (**a**) Multiple I-V curves of fabricated memristor for 1st, 250th and 500th voltage sweep. (**b**) Stability graph of HRs and LRS for 500 voltage sweeps. (**c**) Multiple I-V curves of fabricated memristor after 1 h, 5 h, and 10 h, respectively. (**d**) Retention graph of HRS and LRS for 14 h.

The fabricated memristor was tested for 500 voltage sweeps to check its electrical endurance and obtained I-V curves for the 1st, 250th and 500th voltage sweep are shown in Figure 5a. It can be easily deduced by looking at these three graphs that the memory device remained highly stable for each voltage sweep, which was further verified by plotting the data of HRS and LRS for all voltage sweeps at V_read_ = 0.3 V as shown in Figure 5b. The bistable resistive states were tunable between HRS = 5.8 MΩ and LRS 2.4 kΩ at a read voltage of V_read_ = 0.3 V with a high switching ratio of 2.5 × 10^3^ achieved, which is enough to distinguish between the On and Off state of a memristor. Figure 5c,d exhibit the device performance of the doped TiO_2_ memristor over a long period of time for more than 10 h. It can be seen from the obtained results of both graphs that the I–V curves and plotted data of HRS/LRS were overlapping each other without any major variation. Hence, it can be easily concluded based on the obtained results of endurance and retention data that the fabricated memristive device was highly stable and repeatable for several voltage sweeps and over a long time. 

These electrical characteristic graphs of the TiO_2_-based memristor prove that data can be easily written and erased from this memory device by applying an extremely low electrical voltage (±0.9 V) owing to the co-doping of Cr-N from the solution–gel method. Addition of these metal–non-metal dopants into the thin film of the TiO_2_ functional layer not only helped to further enhance its conductivity (lowering operating voltage and reducing power losses) but also provided extra trapping sites for the charge carriers, hence helping to store data when the external voltage supply was removed. The conduction mechanism of LRS was based on ohmic behavior because the current and voltage were directly proportional to each other with the value of its slope ~1 (I α V). It was verified that the conduction mechanism for the Off state of this device was based on the classical theory of trap-controlled space charge limited current (TCSCLC) owing to the slope values of double logarithmic I-V curves (I α V^m^). This explanation of conduction mechanism is supported by the already reported memristive behavior of TiO_2_-based memory devices [30].

Initially the device was in HRS and there was no movement of charge carriers when no external voltage was applied; however, after the application of an external electric field, electrons begin to drift from anode to cathode and the device switched from HRS to LRS. These electrons are contributed by two different sources including the metallic Ag electrode and the oxygen vacancies of TiO_2_ functional thin film. The donor role of TiO_2_ is due to the presence of inherent defects in it that are responsible for contributing delocalized electrons. Doping with Cr and N helps in lowering the energy bandgap, thus making the migration of charge carriers easy to move from anode to cathode with less power requirements as shown in Figure 3b. It is also believed that the geometry of active material in the form of nanomaterials plays a vital role in enhancing the mobility and conductivity of charge carriers between electrodes because these zero-dimensional materials provide a much higher surface area [41]. These nanomaterials provide a direct pathway and easy tunneling to charge carriers. It can be concluded that the transition from HRS to LRS was due to the ability of charge carriers to tunnel between trap sites provided by metal–non-metal dopants. Furthermore, the achieved results imply that the metallic electrodes might be acting as the reservoir for Cr-N dopants.

### 3.3. Mechanical Characterization

**Figure 6:**
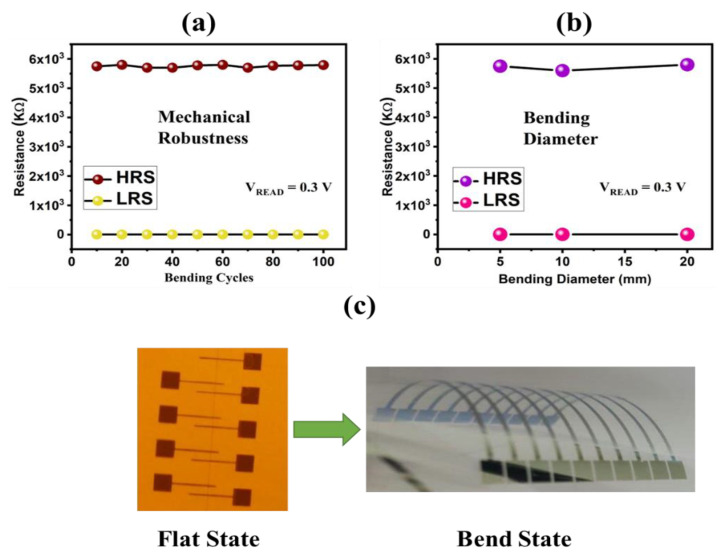
Mechanical robustness of flexible printed memristor. (**a**) Mechanical endurance of doped TiO_2_ memristor for 100 bending cycles at a bending diameter of 5 mm. (**b**) Mechanical endurance of doped TiO_2_ memristor for multiple bending diameters of 20 mm, 10 mm and 5 mm, respectively. (**c**) Optical images of fabricated memristor in flat and bend state.

The fabricated device was fabricated on a flexible PET substrate with the aim to explore its potential use as a wearable memory device of future generations; therefore, we tested its mechanical robustness for 100 bending cycles at a bending diameter of 20 mm. The results shown in Figure 6a show that both HRS and LRS remained highly stable, which is a clear indication that the introduced configuration can withstand this mechanical stress without the formation of any major cracks on its surface. Furthermore, the doped memristor was bent at three different bending diameters ranging from 20–5 mm as shown in Figure 6b. The obtained results have verified that this all-printed nonvolatile memory device can withstand bending at various bending diameters and for multiple bending cycles, which is a highly desirable characteristic for flexible electronics.

## 4. Conclusions

We have doped the function thin film of TiO_2_ with Cr-N using the solution–gel method where the resulting memristive behavior was highly dependent upon the trap sites provided by metal–non-metal doping. The entire device fabrication process was carried out on a flexible substrate by using printing technology. The addition of dopants helped to lower the energy barrier for charge carriers, hence resulting in low operating voltage forming free bipolar resistive switching. The obtained switching ratio was 2.5 × 10^3^ with an operating voltage of ± 1 V. The doped TiO_2_ memristor showed stable nonvolatile memory behavior for more than 500 voltage sweeps and 14 h without any major deviation from the original value. The mechanical robustness of the fabricated memristor was also tested by bending it at multiple bending diameters (5 mm, 10 mm and 20 mm) and for several bending cycles (100 cycles). This work suggests an alternative method of reducing power losses and threshold voltage by lowering energy bandgap for the charge carriers to migrate easily from anode to cathode under the influence of an external electric field.

## Data Availability

The data presented in this study are available on request from the corresponding author.
